# Low Back Pain and Upper-Extremity Musculoskeletal Disorders in French Postal Workers Driving Light-Duty Vehicles for Mail and Parcel Delivery

**DOI:** 10.3390/ijerph20032509

**Published:** 2023-01-31

**Authors:** Anca Radauceanu, Michel Grzebyk, Stéphanie Boini, Mathieu Dziurla, Jean-Jacques Atain-Kouadio, Agnès Aublet-Cuvelier

**Affiliations:** 1 Department of Epidemiology, French Research and Safety Institute for the Prevention of Occupational Accidents and Diseases (INRS), 1, Rue du Morvan, CS 60027, CEDEX, 54519 Vandoeuvre-lès-Nancy, France; 2 Working Life Department, French Research and Safety Institute for the Prevention of Occupational Accidents and Diseases (INRS), 1, Rue du Morvan, CS 60027, CEDEX, 54519 Vandoeuvre-lès-Nancy, France

**Keywords:** musculoskeletal disorders, light-duty vehicle, occupational driving, risk factors, work organization

## Abstract

Occupational driving of light-duty vehicles (LDVs) became increasingly important in parcel delivery faced with the explosive growth of e-commerce. Since musculoskeletal disorders (MSDs) represent the most reported driving-related health problem, we aimed to analyze the risk of low back pain (LBP) and upper-extremity musculoskeletal disorders (UEMSDs) associated with driving LDVs for parcel delivery. In 306 postal workers exposed to driving and 100 unexposed workers, information on occupational driving, physical/psychosocial constraints, and work organization were collected via a questionnaire. MSDs were assessed using the Nordic Questionnaire, 14 additional questions regarding LBP, and a standardized clinical examination for UEMSDs. Statistical modeling consisted of multivariable logistic regression for UEMSDs and the item response theory approach for LBP. UEMSDs were associated with the distance of rural rounds and inversely associated with urban/mixed delivery rounds. Handling heavy loads was associated with LBP, and high physical demands during delivery rounds were related to MSDs. Karasek dimensions and mobbing actions were associated with MSDs. Work recognition, driving training, using an automatic gearbox, and the utilization of additional staff during peak periods were inversely associated with MSDs. Our results suggest that the distance driven in rural settings and high physical demands were associated with MSDs, while some organizational factors could protect from MSDs.

## 1. Introduction

Driving for work on public roads represents an increasing occupational hazard. The 2017 French SUMER survey reported that >25% of employees drove motor vehicles for occupational purposes, while 13% of employees drove > 20 h weekly [1]. The growth of e-commerce [2] and the tertiary sector suggests a progressive increase in the number of these employees for whom driving constitutes, in contrast to professional drivers, only a part of their working day [3].

Other than heavy vehicles driven by “professional drivers”, light-duty vehicles (gross vehicle weight ≤ 3.5 tonnes) are particularly suitable for delivery services because of their flexibility of use. In 2011, the French fleet of professional LDVs consisted of 3.4 million units, and most of them were vans (70%). Motor vehicles represented 20%, and the third most frequent type was vehicles with platforms (4%). One in five professional uses of LDVs was for packages/mail delivery, tenfold higher than with heavy vehicles [4].

The most frequent driving-related health problems are musculoskeletal disorders (MSDs), which are otherwise globally prevalent and costly multifactorial diseases [5]. In France, MSDs represented 90% of occupational diseases and counted for more than 3/4 of repair costs of all occupational diseases in 2016, and figures have remained roughly stable since then [6].

Compared to the professional driving of heavy vehicles [7], the driving of LDVs has received less investigation and focus on the specific occupations (salespeople, taxi drivers, craftspeople) associated most frequently with LBP related to driving time/distance, trunk movements, constrained sitting, and low decision latitude [8,9,10,11]. In a study conducted with pharmaceutical sales representatives, 57% reported low back symptoms in the last 12 months, with prolonged driving, sitting/working in the car, and manual handling as risk factors [12].

To our knowledge, no study about the impact of driving LDVs on MSDs has been carried out in the delivery services of mail/parcels. Moreover, it is difficult to extrapolate the results related to specific occupations involving LDVs as a work tool in the messaging sector. At the time of this study, the number of employees in the French postal company (La Poste group) involved about 6000 operators exclusively driving LDVs for the delivery of parcels in urban/semi-urban settings, about 9000 operators in rural settings delivering parcels by using LDVs among other activities, and about 81,000 operators delivering mail and parcels by foot or using 2-, 3-, or 4-wheelers. An exploratory activity analysis within this company [13] showed that the use of LDVs for postal delivery was only one element in a broader framework that includes phases surrounding delivery rounds, i.e., sorting and loading mail/parcels before and activities at the postal center while returning from rounds. Moreover, the delivery process itself was composed of a repeated series of actions: driving and maneuvering the vehicle, short or long stops, and exiting the driver’s compartment for the delivery activities. The order of the actions performed varied depending on the area of the delivery round, with workers driving short distances for urban/suburban rounds exiting the driver’s compartment half the time and those with rural rounds driving longer distances remaining behind the wheel for longer periods. The majority of subjects included in this exploratory study reported musculoskeletal pain, particularly in the upper limbs and the lower back. Physical constraints (with fragmented driving, frequent exits from the vehicle, climbing up the loading area, handling heavy loads, and long walks), as well as psychological pressures (imputable to heavy traffic, parking problems, and short periods of downtime), are factors that may explain the frequency of MSDs.

The low number of subjects and the self-assessment of musculoskeletal problems demand cautious interpretation; however, this work analysis was hypothesis-generating to carry out a study aimed at investigating the association between driving LDVs for mail/parcel delivery and musculoskeletal disorders and to evaluate the role of work organization in this relationship.

## 2. Methods

### 2.1. Study Population

A cross-sectional study was conducted with employees driving LDVs for mail/parcel delivery ≥ 4 days weekly within the historical French postal company. To control for exposure to other physical constraints unrelated to driving, employees having to engage in the activities of either manually handling loads from 1.5 h to 4 h daily inside the postal center or the walking delivery of mail were also included. All subjects had seniority in their current job (i.e., ≥2 years of experience), and they had not been regraded internally due to health problem. The processing of the collected information has been the object of a declaration to CCTIRS and CNIL, the French Data Protection Authorities. The study population was recruited on a volunteer basis, and each subject provided written informed consent to participate in this study.

### 2.2. Data Collection

Data collection was carried out between 2015 and 2016 by volunteer occupational physicians. Each worker who underwent the compulsory annual medical examination was proposed to participate in this study on a voluntary basis.

### 2.3. Health Outcomes

The appraisal of MSDs began with a self-administered questionnaire based on the French version of the Nordic questionnaire to evaluate regional, nonspecific musculoskeletal symptoms [14].

Low back pain (LBP) was assessed further using specific items in the self-administered questionnaire that were based on published literature [15,16,17,18]. Overall, 14 items allowed for the exploration of LBP within a past time frame (including the total duration of LBP, lowest/highest/at present time pain intensity levels, pain localization and radiation in the lower limbs related to radicular symptoms, use of health care for LBP via medical consultations, pain medication, and sick leave during past 12 months) and within a more recent time frame (including LBP within the past 4 weeks, LBP within the past 7 days, and the intensity of pain while filling out the questionnaire).

Upper extremity musculoskeletal disorders (UEMSDs) were assessed using the standardized clinical evaluation SALTSA [19] for rotator cuff syndrome, lateral epicondylitis, De Quervain’s disease, and carpal tunnel syndrome with three degrees of severity (latent, symptomatic, and clinically proven forms).

### 2.4. Risk Factors of MSDs

#### 2.4.1. Personal Factors

Information was rated on subjects’ sociodemographics, hand dominance, leisure physical activities, smoking, weight, height, and medical history of diabetes mellitus, thyroid disorders, and upper limb and lower back musculoskeletal disorders.

#### 2.4.2. Occupational Exposures

As no specific, validated tool for the assessment of exposure to occupational driving was identified from the literature [20], potential musculoskeletal risk factors were collected using a self-administered questionnaire that had been designed on the basis of the exploratory activity analysis study [13]. The assessment of occupational factors included driving-related factors, physical constraints unrelated to driving, and psychosocial factors. The work organization was appraised at two levels: the worker level, using the above-mentioned questionnaire, and the postal center level, using a questionnaire filled out by its manager.

The driving-related risk factors comprised factors related to the vehicle (whether it was an electric, hybrid, or thermal vehicle and had an automatic or nonautomatic gearbox) and to the delivery round (the type of area served (urban, rural, or mixed), round distance and duration, and time spent driving).

Other physical constraints were related to the work phases surrounding the delivery rounds, such as the sorting and preparation of mail/parcels, the loading and organization of the vehicle, and the delivery process itself. These physical factors included handling loads > 3 Kg, the repetitiveness of handling loads < 3 Kg, and perceived physical demands during the work shift. These latter factors were assessed by means of the Borg rating scales for global perceived exertion (20-RPE, which graduated from 6 “no exertion at all” to 20 “maximal exertion”) and ratings for regional category ratio (CR-10, which graduated from 0 “nothing at all” to 10 “extremely strong”) [21].

Psychosocial factors were recorded through Karasek’s Job Content Questionnaire (from the demand-control model) [22], namely psychological demand, decision latitude, job strain (high psychological demand and low decision latitude), and isostrain (job strain and low social support). The Leymann Inventory of Psychological Terror was used to assess mobbing actions and denial of esteem [23]. Ad-hoc questions were used to explore work recognition and ethical conflicts (undertaking tasks that the worker disapproves of).

Information on work organization was collected at two levels. At the worker level, working schedules, overtime hours, workday pauses, multitasking, owning the workstation, new work organization, assigned objectives, premiums during the last 2 years, and driver training in the past 5 years were assessed. At the center level, information on the new work organization, objectives, the control of employees, and staff management during peak periods was collected.

### 2.5. Statistical Methods

Descriptive results were computed for all subjects and separately for exposed and unexposed groups.

**Figure 1:**
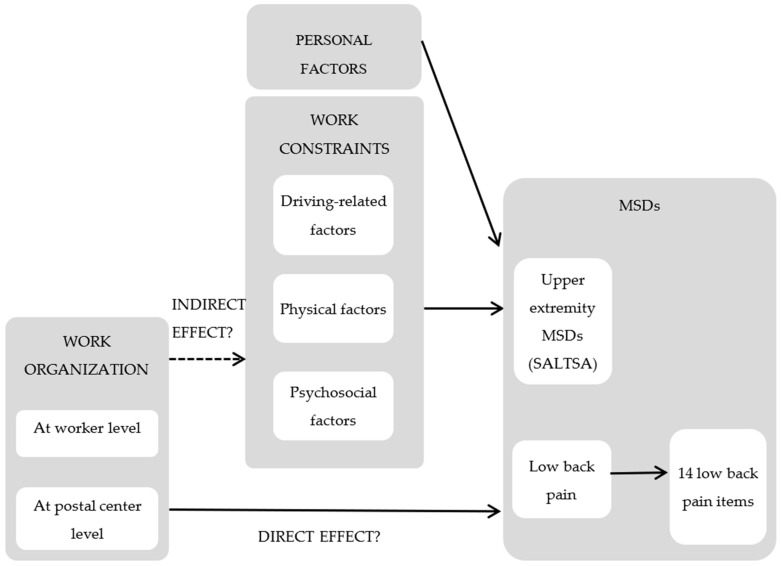
Frame model of the relationships between work situation (work constraints and organization) and musculoskeletal disorders (MSDs) while using light-duty vehicles for delivering mail and parcels.

Analyses were conducted to separately test the effect of driving on UEMSDs and LBP in models including work constraints and personal factors as independent variables, as shown in the conceptual model (Figure 1). These analyses were conducted separately for men and women because of sex-related differences in musculoskeletal health [24].

We considered the following personal factors: age, body mass index, height (for LBP only), history of diabetes mellitus and thyroid disorders, and prior history of UEMSDs/LBP > 2 years.

The work constraints consisted of driving-related, physical, and psychosocial factors (Figure 1). The driving-related factors considered were the type of area served, duration, and distance of the delivery rounds. The physical factors were the time spent handling loads (>3 Kg and repetitively <3 Kg), high global perceived exertion (20-RPE ≥ 14) during sorting mail/parcels, loading the vehicle, and the delivery process, and high regional perceived exertion (CR-10 ≥ 4) for the lower back, shoulders/arms, elbows, and hands during the delivery process. The psychosocial factors included psychological demand, decision latitude, job strain, mobbing actions, work recognition, and undertaking tasks that the worker disapproves of.

As the duration and distance of the delivery round may be correlated on the one hand, and because of the intrinsic relationships between psychological demand, decision latitude, and job strain on the other hand, 4 models, including a combination of these 2 sets of factors and the remaining factors, were tested. The best model, in terms of Akaike Information Criteria (AIC) [25], was thereafter selected for each health outcome and each gender.

For UEMSDs, given the small number of rated cases, the four UEMSDs under study were merged, whatever the laterality, into a single outcome with the most severe clinical form rated (absence < latent < symptomatic < clinically proved), referred to as « UEMSDs » in the remainder of the text. The relationships between driving-related and physical factors and UEMSDs were assessed through ordered logistic regression models adjusted for personal and psychosocial factors.

For LBP, the relationships between the exposure variables and the specific 14 questionnaire items exploring LBP were assessed using structural equation modeling (SEM) [26] based on the conceptual model (Figure 1):

- low back pain is represented by a single continuous variable, with higher values representing worse impairment by convention. This variable cannot be measured directly and is represented by a continuous latent variable;
- the higher the latent variable, the higher the participants’ propensity to rate each of the 14 items at a high level. The relationship between the latent variable and each of the 14 items is modeled by a generalized linear model, with a link function depending on the nature of the item (logistic link for binary items, ordered logistic for ordered categories, and linear for quantitative items);
- the relationship between the physical constraints and the latent variable was assessed through multiple linear regression models adjusted for personal and psychosocial factors. The value of the variance of the residual error of the latent variable was set to 1 to fulfill identifiability.

In this approach, the relevant parameters were the coefficients in the multiple linear regression models between the exposure variables and the latent variable. Positive values or negative values highlighted risk factors or protective factors of LBP, respectively. These models were estimated using the gsem command for STATA software [27].

The relationships between the MSDs and the work organization were assessed based on the conceptual model (Figure 1). In this model, the relationships between MSD and work organization were of two kinds: indirect relationships corresponding to paths from a work organization factor to the outcome through individual occupational factors (physical constraints and psychosocial factors) and direct relationships corresponding to paths from a work organization factor to the outcome without passing through any occupational factors. We implemented a two-step procedure to identify which organizational factor had a direct or indirect effect on the MSDs in relation to the work constraints under study). In the first step, we selected organizational factors using a backward-selection process, adjusted for personal factors omitting work constraints, with a significance level of 0.20 indicating removal from the model. In the second step, the selected organizational factors, the work constraints, and the personal factors were included in the final model. For this model, the statistical significance of the organizational factors indicated a direct relationship with MSDs, independent of the individual occupational factors considered, while statistical insignificance indicated an indirect relationship totally mediated by the work constraints considered. In other words, the statistically insignificant factors indicated an indirect relationship to MSDs through work constraints, which were, in turn, associated with MSDs (Figure 1).

In all models, for both health outcomes, a positive regression coefficient indicated a risk factor for MSDs. In the case of logistic regression, the relative risk could be computed by exponentiating the regression coefficient, but this was not relevant with linear regression. Therefore, the regression coefficients are presented in the results to unify the presentation for both low back pain and UEMSDs. Statistical significance was defined as *p* < 0.05. Analyses were performed using the STATA statistical software (V. 15, StataCorp LLC: College Station, TX, USA).

## 3. Results

### 3.1. General Characteristics

Data collection was performed by 30 (out of 146) volunteer occupational physicians who participated for anywhere from 1 day to 19 months (mean = 7 months) and collected data from between 1 and 45 workers (mean 13 workers per physician). Overall, the study population consisted of 406 full-time and permanent workers belonging to 143 postal centers. Among them, 306 were exposed to driving LDV and 100 were unexposed; specifically, those unexposed were 41 walking postmen and 59 workers handling loads inside postal centers. Personal characteristics, such as sociodemographics and medical history, are presented in Table 1.

### 3.2. Occupational Exposures

The main personal occupational factors related to the driving of light vehicles, physical constraints, psychosocial factors, and work organization are presented in Table 1. Information on center-related organizational factors was collected in 62% (88 out of 143) of postal centers and is shown in Supplementary File S1.

### 3.3. Prevalence of LBP and UEMSDs

#### 3.3.1. Self-Reported Symptoms

Almost one-quarter of the study population reported upper extremity symptoms over a period longer than 30 days or on a daily basis in the last 12 months. On a visual analog scale ranging from 0 to 10, the mean intensity of upper limbs symptoms while filling out the questionnaire ranged from 3.1 to 4.1 in men and 3.4 to 4.5 in women, whereas for LBP, it was 3.7 in men and 4.7 in women. One-third of those exposed to LDVs and one-third of the unexposed subjects rated pain levels as ≥6 for LBP during the last 12 months. LBP radiation in the lower limbs was reported by 29% of the whole population. Full details are presented in Table 2 (prevalences of musculoskeletal symptoms were taken from the Nordic questionnaire) and Table 3 (14 LBP items).

#### 3.3.2. Clinically Diagnosed UEMSDs

A total of 71 subjects experienced 150 UEMSDs that were rated, whatever the laterality, in the clinical form (latent, symptomatic, or diagnosed) and as muscular disorders (rotator cuff syndrome, lateral epicondylitis, De Quervain’s disease, and carpal tunnel syndrome). The prevalence of at least one clinically diagnosed UEMSD was 10.5% in exposed and 9% in unexposed subjects. In the whole population, the most clinically diagnosed UEMSD was rotator cuff syndrome (6.6%), followed by lateral epicondylitis (3.4%), carpal tunnel syndrome (1.5%), and De Quervain’s disease (0.7%) (Table 4).

### 3.4. Relationship between Work Constraints and MSDs

In the analyses of LBP, the factor loadings between the latent variable and each of the LBP items were all positive (*p* < 0.05), indicating that the latent scale was consistent with health impairment in the low back.

In men and women, the models with the duration of the delivery rounds were selected for LBP, whereas the models with the distance of the delivery rounds were selected for UEMSDs.

Table 5 shows work constraints associated with LPB or UEMSDs in the selected models adjusted for personal factors separately for men and women.

In men as well as in women, LBP was positively associated with higher levels of lower back physical demand during delivery, and negatively associated with the duration of rural rounds. In addition, LBP in women was positively associated with handling heavy loads during a work shift and psychological demands and negatively associated with decision latitude.

In men and women, UEMSDs were positively associated with the distance of rural rounds (although not statistically significant in women) and negatively related to the urban/mixed type of area served during the delivery round. In men, UEMSDs were positively associated with high physical demands (global and elbow) during delivery and negatively associated with psychological demands, decision latitude, and work recognition. In women, UEMSDs were strongly positively associated with workplace mobbing actions.

### 3.5. Relationship between Work Organization and MSDs

The results of the selection process of the organizational factors are reported in Supplementary File S2. At the worker level, the selected factors were those related to new work organization, work schedule and pauses, the round holder, type of vehicle, training courses, operators’ premiums, and objectives. At the postal center level, the selected factors were those related to new work organization, workforce management during peak periods, operators’ control, and objectives evolution.

Associations between the selected organizational factors and MSDs in models, adjusted for personal factors and work constraints, are shown in Table 6 (Model I for the worker level and Model II for the center level). The organizational factors with statistically significant parameters were considered as having a direct effect on MSDs, while those that were nonsignificant were considered as having an indirect effect mediated by the work constraints. The organizational factors having a direct effect on MSDs differed with gender and the MSDs. Thus, LBP was negatively associated with having an automatic gearbox, the control of operators, and belonging to a center using additional staff during peak periods in men, and with receiving premiums in the last 2 years in women. UEMSDs were positively associated with flexible working time during peak periods and negatively associated with having an automatic gearbox (although not statistically significant) in men, and negatively associated with driving training in the past 5 years in women. Surprisingly, LBP in men and UEMSDs in women were positively associated with the use of electric/hybrid vehicles.

## 4. Discussion

### 4.1. Main Findings

This study highlighted the factors associated with MSDs (LBP and UEMSDs) in workers driving LDVs for mail/parcel delivery.

For men and women, risk factors for UEMSDs were mostly related to the type of served area, with a higher risk of UEMSD in rural rounds compared to urban/mixed rounds, and a positive association with the distance of rural rounds, while its duration was negatively associated with LBP.

Handling heavy loads during the workday in women and high perceived physical demands during delivery in men and women were positively associated with MSDs. We found associations between psychological demand, decision latitude, and MSDs in both men and women. Furthermore, mobbing actions were positively associated with UEMSDs in women and work recognition was negatively associated with UEMSDs in men.

Organizational factors that were negatively related, and thus, potentially protective against MSDs were: using a vehicle fitted with an automatic gearbox in men, following driving courses in women (UEMSDs), and having additional staff during peak periods in men (LBP). On the contrary, potential risk factors were flexible working time during peak periods in men (UEMSDs) and using electric/hybrid vehicles in men and women.

### 4.2. Comparison of Prevalence of MSDs

Compared to those reported in the overall working population sample of the Pays de la Loire survey [30,31] the prevalences of MSDs from the Nordic questionnaire were generally higher in our study, except for those relating to the LBP lasting > 30 days during the last 12 months that were lower (15% vs. 28% for men; 14% vs. 33% for women). The major differences were for LBP in men during the preceding week (31% vs. 28%) and for UEMSDs in women lasting > 30 days (35% vs. 19%) and during the last week (46% vs. 35%).

The clinically proven UEMSDs rates from the SALTSA evaluation were lower than those reported in the Pays de la Loire survey (10% vs. 13%) except for the lateral epicondylitis (3.4% vs. 2.2%), whereas the most reported UEMSD was rotator cuff syndrome in both studies (6.6% vs. 7.8%).

Among professional drivers, a recent international review found a lower prevalence of LBP compared to drivers in our study (53% vs. 60%) [5].

### 4.3. Comparison of Risk Factors for MSDs with Previous Literature

Few studies analyzed musculoskeletal risk within mail/parcel services. We found that high physical demands were associated with LBP, especially in women, in accordance with a prospective study including postal workers that found a risk of chronic LBP related to heavy lifting and walking in women but not in men [32]. The authors suggest that women and men may have differences in work exposures, different perceptions of pain, as well as differences related to biological, social, and psychological aspects. Consistently, in a large cohort of employed Swedish residents, a higher physical workload was a risk factor for early-age retirement associated with previous sickness absence for back pain among women only [33].

We found for men as well as for women that risk factors for MSDs, and especially for UEMSDs, were mostly related to the type of area served, with a higher level of risk in rural rather than in urban/mixed settings. Compared to rural rounds, urban/suburban rounds involve driving short distances with exits from the driver’s compartment half the time [13]. This may explain the longer time spent driving in rural rounds, whilst driving time was reported as a risk factor of LBP (>4 h/day in taxi drivers [34] or ≥10 h/week in commercial travelers [35]). Regarding the postal sector, we did not find published data except those from a retrospective survey of retired postal workers reporting that MSDs were associated with occupational driving time > 4 h/day [36]. Nevertheless, in our study, the duration of the delivery round was negatively associated with LBP, no matter the type of area served, while the distance of rural rounds was positively related to UEMSDs. We assume that the declarative duration of the delivery round could be more subjective than its distance, since it may depend on traffic issues [13].

We found for men as well as for women that risk factors for MSDs, and especially for UEMSDs, were mostly related to the type of area served, with a higher level of risk in rural rather than in urban/mixed settings. Compared to rural rounds, urban/suburban rounds involve driving short distances with exits from the driver’s compartment half the time [13]. This may explain the longer time spent driving in rural rounds, whilst driving time was reported as a risk factor of LBP (>4 h/day in taxi drivers [34] or ≥10 h/week in commercial travelers [35]). Regarding the postal sector, we did not find published data except those from a retrospective survey of retired postal workers reporting that MSDs were associated with occupational driving time > 4 h/day [36]. Nevertheless, in our study, the duration of the delivery round was negatively associated with LBP, no matter the type of area served, while the distance of rural rounds was positively related to UEMSDs. We assume that the declarative duration of the delivery round could be more subjective than its distance, since it may depend on traffic issues [13].

Epidemiological studies were carried out with no distinction between specific activities of postal workers [37,38,39] and indicated that UEMSDs and LBP were associated with carrying loads [39,40]. In Denmark, the one-year prevalence rate for LBP in active postal workers was higher (62% in women, 52% in men) than in the general population [41]. A study conducted among retired English mail workers showed that work-related MSDs persisted even after the exposure ended, and varied between 20% and 50% for shoulders/hands, hips/knees, and the lower back one month after the end of exposure [36]. In this post-retirement group, associations were also found between MSDs and carrying loads, awkward postures, and occupational driving > 4 h daily [36]. In a study on the multifactorial determinants behind sick leave in Swedish mail carriers, the strongest relationship was found with anxiety about workplace reorganization, followed by carrying heavy loads [42]. In a New Zealand survey of a population including postal workers (whose primary job was sorting the mail), nurses, and office workers, MSDs of the lower back, shoulders, and wrists/hands were associated with physical demands, job strain, and job dissatisfaction [40].

Our study brought out relationships between the psychosocial scales of Karasek’s demand-control model and MSDs in men and women, in accordance with previous literature [43,44]. Compared to employees driving for work in the 2010 SUMER survey, workers driving LDVs in our study exhibited higher instances of job strain (30% vs. 15%) and isostrain (19% vs. 10%) [1]. A recent review reported job strain as an independent risk factor of musculoskeletal pain [45]. Psychosocial factors linked to work, such as strong psychological pressure associated with a weak level of autonomy, give rise to situations of “workplace tension or job strain”, particularly in cases where workers lack support from their hierarchy [44]. Among employed Swedish residents, job strain was consistently associated with the risk of disability pension related to back pain in women but not in men, whereas low job control was associated with this risk among both men and women [33].

We also showed an overall protective effect of work recognition and a strong association of mobbing actions with UEMSDs in women. Less analyzed as a psychosocial stressor of musculoskeletal problems, workplace bullying is increasingly being recognized as a serious issue within the workplace environment and a risk factor for absenteeism [44].

### 4.4. Factors Related to Work Organization

Work organization relates to different patterns of organizational and psychosocial variables and may influence MSDs [46]. In our study, we collected information on work organization at both the worker and center levels, and we assessed the direct effects of organizational factors. We highlighted the management practices within postal centers during peak periods, consisting of the use of additional staff as a protective factor, whereas flexible working time was positively associated with MSDs. Accordingly, in a recent pooled study, organizational factors, such as overtime work and job rotation, were associated with high biomechanical and psychosocial exposures [47].

We showed that using an automatic gearbox and completing driving courses were potentially protective factors from LBP and UEMSDs, and these results represent new findings to our knowledge. As these organizational factors were directly and negatively related to MSDs, and thus do not pass through work constraints, their implementation within work organization may prevent MSDs.

The results of a recent review and meta-analysis showed that the effects of workplace interventions in workers’ populations affected by LBP led to a significant improvement in clinical outcomes [48]. In our study, we highlighted some potentially protective organizational factors at the worker level and at the company level that could be implemented in an integrated, multilevel, and multidimensional prevention strategy in addition to reducing common physical and psychosocial risk factors of MSDs. Actually, to obtain positive results from ergonomic interventions aimed at preventing work-related musculoskeletal disorders, strong support for an intervention program from co-workers, supervisors, and head management was underlined [49]. A recent study suggested the importance of targeting work organization at different levels when designing interventions to reduce low back pain and sickness absence in eldercare working in many wards across some nursing homes [50]. Whether low back pain was associated with increased worker-level quantitative demands, sickness absence was associated with nursing-home quantitative demands (but not at the worker-level or ward level). Therefore, the interventions to reduce sickness absence should target nursing homes, thus including all workers, contrary to specific “at risk” wards or workers.

### 4.5. Strengths and Limitations of the Study

The health assessment included a standardized evaluation of UEMSDs [19]. In the absence of a standardized procedure, we explored LBP with a specific questionnaire in an IRT-like model.

The physical workload was self-reported and any objective measure of physical demands was performed in the study. Nevertheless, self-reported subjective measures of physical hazards seem to more accurately assess some physical demands, such as postures involving heavy exertion and isometric activities [51]. On the other hand, the workers’ perception of exertion may be affected by worker-related psychosocial factors and a quantification of exertion assessment by means of physiological or ergonomic approaches should be encouraged [52]. Lastly, the exploratory work analysis that we carried out allowed the assessment of self-reported work exposures with potentially less bias [53].

As the study population included workers that had not been regraded internally due to health problems, we considered, in statistical models, prior history of UEMSDs/LBP > 2 years but not past occupational exposure to driving or to risk factors of MSDs.

Due to the transversal design of our study, it is not possible to infer causality, some associations may derive from reverse causality. Although the study was proposed to each eligible worker and the occupational physicians completed any selection at inclusion, information on the participation rate was not collected. In addition, conducted within a single company, it makes it difficult to extrapolate the results to a wholly messaging service. Likewise, the issues of selection bias and statistical power support a cautious interpretation of the results.

## 5. Conclusions

Distance and rural settings of delivery rounds were associated with UEMSDS, while high perceived physical demands were associated with LBP in both genders and with UEMSDs in men. For both genders, demand—control model dimensions were associated with MSDs, whereas work recognition seems protective from UEMSDs. Mobbing actions were a strong risk factor for UEMSDs in women. Targeting work organizations at different levels and lowering work exposures should be integrated into global prevention approaches.

As the study was conducted within the French national postal service, its findings could be generalized to the parcel delivery sector in general or other geographical regions after verifying that work characteristics are similar. Otherwise, further specific studies have to be implemented preferably prospectively designed for analyzing causality.

Preventing MSDs in activities of delivering mail/parcels by using LDV requires a multilevel and multidimensional integrated approach targeting co-workers, supervisors, and top management. This study suggests that, in these occupational activities, MSD risk factors are found among driving-related characteristics as well as among features that are not directly related to driving. The use of light vehicles fitted with an automatic gearbox for delivery rounds as well as completing driving training for employees possibly play a protective role against MSDs. In addition, the prevention of MSDs should focus on employees driving in a rural setting and for long distances.

The workforce management during peak period should prioritize the use of additional staff more than overtime hours of permanent workers. The implementation of a positive work climate should target increasing work recognition and decision latitude, and lowering workplace bulling.

It should be noted that the use of electric vehicles appeared as a risk factor in this study, whereas the use of electric vehicles is becoming more widespread; further studies should be implemented to specifically address this issue.

## Figures and Tables

**Table 1:** Main personal characteristics and occupational factors of the study population exposed to driving light-duty vehicles and unexposed subjects. Abbreviations: LDV: light-duty vehicle; UEMSDs: upper extremity musculoskeletal disorders; LBP: low back pain; m (SD): mean (standard deviation).

	Altogether (*n* = 406)	Exposed (*n* = 306)	Unexposed (*n* = 100)
**Personal characteristics**			
Age, years (m(SD))	44 (9.3)	44 (9.4)	47 (8.7)
<30 y. %	7	9	4
30–39 y. %	24	26	17
40–49 y. %	36	35	41
50–59 y. %	30	28	35
≥60 y. %	2	2	3
Men (%)	64	63	69
Height, cm (m(SD)) [28,29]	171 (9.2)	171 (9.4)	171 (8.7)
Men > 180 cm. %	23	27	14
Women > 170 cm. %	12	11	16
Body mass index (BMI), Kg/cm^2^ (m(SD))	26 (4.4)	26 (4.6)	25 (3.7)
BMI < 25, %	50	48	55
BMI ≥ 25, %	51	52	45
Right-handed, %	87	88	87
Tobacco consumption			
Never, %	56	58	51
Current or former, %	44	41	49
Leisure time physical activities			
Sport, %	46	49	39
Gardening and DIY, %	50	51	44
Education, %			
No diploma	5	6	5
Lower vocational	39	39	39
Medium-high	52	52	54
Prior history of at least one of the UEMSDs, %	53	54	50
Prior history of LBP	57	59	53
Diabetes mellitus, %	2	2	1
Thyroid disorders, %	3	3	4
**Occupational factors**			
Length of current job, years (m(SD))	13 (9)	13 (9)	11 (9)
State hired, %	37	34	47
Permanent work contract, %	63	36	53
***Driving-related factors***			
Electric vehicle, %	-	25	-
Automatic gearbox	-	20	-
Assigned vehicle, %	-	66	*-*
Postal round characteristics			
Urban area, %	-	24	-
Rural area, %		39	
Mixed area, %		35	
Distance of round, km (m(SD))		42 (25.5)	
Duration of round, hours, %			
<2 h		7	
2–4 h		53	
4 h		36	
Time spent driving, % of round duration (m(SD))	-	61 (20.4)	-
***Physical constraints***			
Handling loads weighing more than 3 Kg > 4 h/day, %	14	9	27
High ^1^ repetitively handling loads weighing less than 3 Kg > 4 h/day, %	17	12	30
High perceived physical demand rated with Borg scales ^2^			
Global physical demand (20-RPE) ≥ 14 during sorting/preparation of the delivery, %	15	13	27
Global physical demand (20-RPE) ≥ during loading/organization of mail/parcels, %	16	17	10
Global physical demand (20-RPE) ≥ 14 during delivery, %	30	31	27
Low back category ratio (CR-10) ≥ 4 during delivery, %	61	61	61
Shoulder/arm category ratio (CR-10) ≥ 4 during delivery, %	54	53	58
***Psychosocial factors***			
Karasek model scores (median (range))			
Psychological demand	22 (9–36)	22 (9–36)	22 (12–34)
Decision latitude	63 (28–88)	63 (28–88)	62 (34–88)
Job strain ^3^, %	29	30	25
Isostrain ^4^, %	17	19	14
Current mobbing actions, %	11	10	14
Current denial of esteem, %	10	10	10
Work recognition, %	96	97	93
Undertaking tasks that the worker disapproves of, %	62	63	61
***Work organization***			
Regular schedules, %	76	78	70
Overtime hours, %	77	84	57
Workday pauses, %	71	68	80
Workstation holder, %	75	73	81
Regularly multitasking, %	22	17	40
LDV driving training, %	78	90	41
Concerned by new work organizations, %	62	63	59
Perceived difficult-to-achieve assigned objectives	37	37	37
Premiums during the last 2 years	90	93	80

^1^ More than 2–4 times/min, ^2^ Unexposed group is composed only of employees who carry out foot delivery of mail, ^3^ Job strain corresponds to high psychological demand and low decision latitude (reference value of 27.3% in the SUMER 2010 survey: French medical surveillance of professional risks), ^4^ Isostrain corresponds to job strain and low social support (reference value of 17.25 % in the SUMER 2010 survey).

**Table 2:** Frequency of musculoskeletal symptoms experienced by study subjects in the upper-extremity and back areas (number of subjects (%)). Abbreviations: UEMSDs: upper extremity musculoskeletal disorders.

	Symptoms in the Last 12 Months			Symptoms > 30 Days in the Last 12 Months, Cumulatively or Daily			Symptoms in the Last 7 Days		
	Altogether	Exposed	Unexposed	Altogether	Exposed	Unexposed	Altogether	Exposed	Unexposed
**UEMSDs**									
Neck/Cervical region	131 (32.3)	93 (30.4)	38 (38.0)	38 (9.4)	24 (7.8)	14 (14.0)	80 (19.7)	56 (18.3)	24 (24.0)
Shoulder/Arm	158 (38.9)	123 (40.2)	35 (35.0)	62 (15.3)	48 (15.7)	14 (14.0)	94 (23.2)	70 (22.9)	24 (24.0)
Elbow/Forearm	91 (22.4)	69 (22.6)	22 (22.0)	28 (6.9)	24 (7.8)	4 (4.0)	58 (14.3)	44 (14.4)	14 (14.0)
Wrist/Hand	100 (24.6)	73 (23.9)	27 (27.0)	26 (6.4)	17 (5.6)	9 (9.0)	60 (14.8)	40 (13.1)	20 (20.0)
Fingers	79 (19.5)	53 (17.3)	26 (26.0)	20 (4.9)	14 (4.6)	6 (6.0)	44 (10.8)	29 (9.5)	15 (15.0)
Upper extremity (at least one symptom)	227 (55.9)	172 (56.2)	55 (55.0)	96 (23.7)	72 (23.5)	24 (24.0)	154 (37.9)	115 (37.6)	39 (39.0)
**Back pain**									
Upper back	103 (25.4)	75 (24.5)	28 (28.0)	29 (7.1)	18 (5.9)	11 (11.0)	58 (14.3)	41 (13.4)	17 (17.0)
Low back	238 (58.6)	182 (59.5)	56 (56.)	59 (14.5)	40 (13.1)	19 (19.0)	127 (31.3)	99 (32.4)	28 (28.0)
Upper or low back	248 (61.1)	190 (62.1)	58 (58.0)	69 (17.0)	48 (15.7)	21 (21.0)	159 (39.2)	124 (40.5)	35 (25.0)

**Table 3:** The 14-item questionnaire to assess low back pain within two time frames (last 12 months and recently) in subjects exposed and unexposed to driving. Except for the total length of time of low back pain in the past 12 months, the percentages are calculated excluding the 163 postal workers declaring no low back pain in the past 12 months in the Nordic questionnaire. Abbreviations: N: number of subjects; m(sd) [min; max]: mean (standard deviation) [minimum and maximum values of data].

Variables	Altogether		Exposed		Unexposed	
**In the past 12 months**						
Total length of time of low back pain, N (%)						
no LBP in the past 12 months	163	40.1%	120	39.2%	43	43.0%
<1 day	9	2.2%	7	2.3%	2	2.0%
1 to 7 days	61	15.0%	48	15.7%	13	13.0%
8 to 30 days	46	11.3%	37	12.1%	9	9.0%
more than 30 days	36	8.9%	25	8.2%	11	11.0%
permanently	23	5.7%	15	4.9%	8	8.0%
LBP in the past 12 months but missing frequency	63	15.5%	50	16.3%	13	13.0%
LBP in the past 12 months missing	5	1.2%	4	1.3%	1	1.0%
The most intense, mean (sd) [min ; max]	5.6 (3.0) [0 ; 10]		5.7 (2.8) [0 ; 10]		5.4 (3.4) [0 ; 10]	
The less intense, mean (sd) [min ; max]	1.6 (1.7) [0 ; 7]		1.6 (1.7) [0 ; 7]		1.6 (1.9) [0 ; 6]	
Intensity at the present time, mean (sd) [min ; max]	2.0 (2.3) [0 ; 10]		1.9 (2.3) [0 ; 10]		2.2 (2.1) [0 ; 7]	
Pain localized to the lower back without radiating in the lower limbs, N (%)						
no	110	45.3%	82	44.1%	28	49.1%
yes	122	50.2%	93	50.0%	29	50.9%
missing	11	4.5%	11	5.9%	0	0.0%
Pain that radiates above the knee, N (%)						
no	199	81.9%	153	82.3%	46	80.7%
yes	33	13.6%	22	11.8%	11	19.3%
missing	11	4.5%	11	5.9%	0	0.0%
Pain that radiates below the knee, N (%)						
no	144	59.3%	110	59.1%	34	59.6%
yes	88	36.2%	65	34.9%	23	40.4%
missing	11	4.5%	11	5.9%	0	0.0%
Other types of low back pain (discomfort, numbness, aching), N (%)						
no	180	74.1%	138	74.2%	42	73.7%
yes	52	21.4%	37	19.9%	15	26.3%
missing	11	4.5%	11	5.9%	0	0.0%
Medical consultation (once, at least), N (%)						
no	108	44.4%	86	46.2%	22	38.6%
yes	121	49.8%	87	46.8%	34	59.6%
missing	14	5.8%	13	7.0%	1	1.8%
Using pain medication (once, at least), N (%)						
no	96	39.5%	78	41.9%	18	31.6%
yes	133	54.7%	95	51.1%	38	66.7%
missing	14	5.8%	13	7.0%	1	1.8%
Sick leave (once, at least), N (%)						
no	163	67.1%	127	68.3%	36	63.2%
yes	66	27.2%	46	24.7%	20	35.1%
missing	14	5.8%	13	7.0%	1	1.8%
**Recent time frame (in the last 4 weeks and earlier)**						
Low back pain in the past 4 weeks, N (%)						
no	105	43.2%	80	43.0%	25	43.9%
yes	128	52.7%	97	52.2%	31	54.4%
missing	10	4.1%	9	4.8%	1	1.8%
Low back pain in the past 7 days, N (%)						
no	104	42.8%	77	41.4%	27	47.4%
yes	127	52.3%	99	53.2%	28	49.1%
missing	12	4.9%	10	5.4%	2	3.5%
The intensity of pain while filling out the questionnaire, mean (sd) [min ; max]	2.2 (2.6) [0 ; 10]		2.2 (2.6) [0 ; 10]		2.2 (2.9) [0 ; 9]	

**Table 4:** Frequency of clinically diagnosed upper extremity musculoskeletal disorders (UEMSDs) according to the exposure group.

UEMSDs (%)	Altogether (*n* = 406)	Exposed (*n* = 306)	Unexposed (*n* = 100)
Rotator cuff syndrome	6.6	6.9	6.0
Lateral epicondylitis	3.4	4.5	1.0
De Quervain’s disease	0.7	1.0	0
Carpal tunnel syndrome	1.5	1.3	2.0
At least one of the 4 UEMSDs	10.1	10.5	9.0

**Table 5:** Occupational factors associated with low back pain (LBP) and clinically diagnosed upper-extremity musculoskeletal disorders (UEMSDs) in workers driving a light-duty vehicle (LDV), expressed by a regression coefficient β (*p*-value) [95% confidence interval] in models adjusted for personal factors. A positive coefficient indicates a risk factor and a negative one indicates a potentially protective factor. Bold figures indicate statistically significant *p* < 0.05. (a) factor is not in the model selected; (b) factor excluded from models of LBP; (c) factor excluded from models of UEMSDs.

	LBP		UEMSDs	
Occupational Factors	Men	Women	Men	Women
	β (*p*) [95% CI]	β (*p*) [95% CI]	β (*p*) [95% CI]	β (*p*) [95% CI]
*Observations*	188	113	177	100
**Driving-related and physical constraints**				
Urban/mixed delivery round vs. rural delivery round	−0.42 (0.555) [−1.80; 0.96]	−0.46 (0.565) [−2.04; 1.11]	−3.28 **(0.041)** [−6.43; −0.13]	−5.95 **(0.032)** [−11.39; −0.51]
Duration of delivery round (per hour)				
in urban/mixed round	−0.22 (0.060) [−0.46; 0.01]	−0.31 **(0.006)** [−0.53; −0.09]	(a)	(a)
in rural round	−0.20 **(0.017)** [−0.37; −0.04]	−0.30 **(0.049)** [−0.60; −0.00]	(a)	(a)
Distance of delivery round. urban/mixed (per km)				
in urban/mixed round	(a)	(a)	−0.01 (0.566) [−0.05; 0.03]	−0.05 (0.084) [−0.11; 0.01]
in rural round	(a)	(a)	0.04 **(0.016)** [0.01; 0.07]	0.06 **(0.053)** [−0.00; 0.12]
High repetitively handling loads < 3 Kg (per hour during work shift)	(b)	(b)	−0.16 (0.252) [−0.42; 0.11]	−0.36 (0.194) [−0.91; 0.18]
Handling loads > 3 kg (per hour during work shift)	0.03 (0.576) [−0.07; 0.13]	0.22 **(0.010)** [0.05; 0.39]	(c)	(c)
Rating global perceived exertion Borg scale (20-RPE) ≥ 14				
during the sorting/preparation of the delivery	−0.54 (0.097) [−1.18; 0.10]	0.04 (0.919) [−0.74; 0.82]	0.67 (0.398) [−0.89; 2.24]	−1.08 (0.475) [−4.05; 1.89]
during the loading/organization of mail/parcels	0.30 (0.285) [−0.25; 0.84]	0.71 (0.070) [−0.06; 1.47]	−1.16 (0.118) [−2.61; 0.29]	2.33 (0.136) [−0.73; 5.39]
during delivery operation	0.35 (0.108) [−0.08; 0.78]	0.16 (0.609) [−0.46; 0.78]	1.24 **(0.031)** [0.11; 2.36]	−0.85 (0.518) [−3.45; 1.74]
Lower back category ratio Borg scale (CR-10) ≥ 4 during delivery	0.50 **(0.020)** [0.08; 0.92]	0.63 **(0.029)** [0.06; 1.19]	(c)	(c)
Shoulder/arm category ratio Borg scale(CR-10) ≥ 4 during delivery	(b)	(b)	0.64 (0.315) [−0.61; 1.88]	0.58 (0.627) [−1.77; 2.94]
Elbow category ratio Borg scale (CR-10) ≥ 4 during delivery	(b)	(b)	1.93 **(0.002)** [0.70; 3.16]	1.54 (0.142) [−0.51; 3.59]
Hand category ratio Borg scale (CR-10) ≥ 4 during delivery	(b)	(b)	−0.38 (0.536) [−1.59; 0.83]	−2.06 (0.106) [−4.56; 0.44]
**Psychosocial factors**				
Psychological demand	(a)	0.09 **(0.007)** [0.03; 0.16]	−0.17 **(0.023)** [−0.33; −0.02]	(a)
Decision latitude	(a)	−0.05 **(0.001)** [−0.08; −0.02]	−0.07 **(0.014)** [−0.13; −0.01]	(a)
Job strain	−0.08 (0.685) [−0.44; 0.29]	(a)	(a)	2.10 (0.072) [−0.19; 4.38]
Work recognition	−0.22 (0.064) [−0.44; 0.01]	−0.06 (0.749) [−0.44; 0.32]	−1.34 **(0.005)** [−2.27; −0.40]	2.05 **(0.026)** [0.24; 3.86]
Undertaking tasks that the worker disapproves of	0.11 (0.449) [−0.18; 0.41]	−0.29 (0.166) [−0.71; 0.12]	−0.22 (0.611) [−1.06; 0.62]	−0.95 (0.201) [−2.40; 0.50]
Mobbing actions	0.32 (0.159) [−0.12; 0.76]	0.08 (0.823) [−0.62; 0.78]	0.25 (0.739) [−1.21; 1.70]	6.74 **(0.002)** [2.46; 11.02]

**Table 6:** Selected organizational factors associated with low back pain (LBP) and clinically diagnosed upper-extremity musculoskeletal disorders (UEMSDs) in workers driving a light-duty vehicle (LDV), expressed by a regression coefficient β (*p*-value) [95% confidence interval] in models adjusted for personal factors and work constraints. A positive coefficient indicates a risk factor and a negative one indicates a potentially protective factor; bold figures indicate statistically significant *p* < 0.05; (a), organizational factors not selected in the stepwise procedure; (b), model unavailable.

	LBP		UEMSDs	
Organizational Factors	Men	Women	Men	Women
	β (*p*) [95% CI]	β (*p*) [95% CI]	β (*p*) [95% CI]	β (*p*) [95% CI]
**Model I. At worker level**				
*Observations*	188	111	177	98
Concerned by new work organizations (yes vs. no)	0.26 (0.070) [−0.02; 0.53]	0.25 (0.337) [−0.26; 0.76]	0.89 (0.070) [−0.07; 1.85]	(a)
Work schedule (vs. regular hours)				
Irregular hours	−0.28 (0.416) [−0.95; 0.39]	(a)	−0.66 (0.499) [−2.58; 1.26]	(a)
Staggered hours	−0.68 **(0.007)** [−1.18; −0.18]	(a)	−0.06 (0.944) [−1.67; 1.55]	(a)
Can take a break (yes vs. no)	(a)	(a)	0.21 (0.734) [−1.02; 1.45]	(a)
Round holder (yes vs. no)	(a)	(a)	1.11 (0.151) [−0.41; 2.63]	(a)
Driver training in the past 5 years (yes vs. no)	(a)	0.20 (0.624) [−0.60; 1.01]	(a)	−11.55 **(0.005)** [−19.62; −3.49]
Automatic gearbox vs. non-automatic gearbox	−0.69 **(0.045)** [−1.37; −0.02]	(a)	−1.70 (0.058) [−3.46; 0.06]	(a)
Electric/hybrid vehicle vs. combustion-powered vehicle	0.57 (0.056) [−0.01; 1.15]	(a)	(a)	6.00 **(0.022)** [0.87; 11.13]
Perceived difficult-to-achieve assigned objectives (yes vs. no)	(a)	(a)	(a)	1.16 (0.350) [−1.27; 3.60]
Premium in the last 2 years (yes vs. no)	(a)	−3.68 **(0.000)** [−5.38; −1.98]	(a)	(a)
Handling loads training in the past 5 years (yes vs. no)	(a)	(a)	−0.45 (0.515) [−1.82; 0.92]	3.86 **(0.029)** [0.38; 7.33]
**Model II. At center level**				
*Observations*	100	69	114	69
New work organization (vs. no new work organization)				
with lunchbreak	(a)	0.71 (0.077) [−0.08; 1.49]	(a)	(b)
without lunchbreak	(a)	−0.04 (0.925) [−0.92; 0.84]	(a)	(b)
Flexible working time during peak periods	(a)	−0.01 (0.987) [−0.78; 0.77]	2.36 **(0.007)** [0.65; 4.07]	(b)
Use of additional staff during peak periods	−0.77 **(0.043)** [−1.51; −0.03]	(a)	−2.10 (0.071) [−4.37; 0.18]	(b)
Evolution towards more demanding objectives during the last 2 years	0.42 (0.130) [−0.12; 0.96]	(a)	(a)	(b)
Operators’ control (computer-based and management-based)	−0.80 **(0.029)** [−1.51; −0.08]	(a)	(a)	(b)

## Data Availability

The data presented in this study should not be shared due to legal issues.

## Supplementary Materials

The following supporting information can be downloaded at: https://www.mdpi.com/article/10.3390/ijerph20032509/s1, Supplementary File S1: Main center-related organizational factors collected by questionnaire from postal centers where participants of the study were recruited. Supplementary File S2: Organizational factors selected in the backward-stepwise selection (*p* < 0.20) adjusted on personal factors.
