# Supplementary material for: Low Back Pain and Upper-Extremity Musculoskeletal Disorders in French Postal Workers Driving Light-Duty Vehicles for Mail and Parcel Delivery
Source: Int J Environ Res Public Health. 2023 Jan 31;20(3):2509. doi: 10.3390/ijerph20032509 (PMC9916289; doi:10.3390/ijerph20032509)
Supplement: Supplementary file 1 [file ijerph-20-02509-s001.zip › Supplementary File S1.pdf]

Supplementary File S1. Main organizational data collected by questionnaire from postal centers where participants of the study were recruited. Abbreviations: m±SD: mean ± standard deviation; min-max: minimum and maximum values of data; LDV: light-duty vehicle.

Postal centers where study population was recruited, number	143
Postal centers response rate to the questionnaire, number (%)	88 (62%)
Subjects included in the study, number of centers (%)	
1-3 subjects	115 (80%)
4-9 subjects	20 (14%)
>10 subjects	8 (6%)
Workforce postal centers	
number of employees, m±SD (min-max)	126.4±135 (3-550)
men, %	48
Type of employment contract, % in postal center (m±SD)	
Permanent work contract	53.3±14.3
Contract of determined length	5.2±6.3
State hired	38.1±15.6
Temporary employment	1.6±3.5
Light-duty vehicle fleet per postal center, number (m±SD)	
Thermal vehicle	31.2±27.4
Electric vehicle	7±8.7
Hybrid vehicle	0
Shared vehicles with other centers	0.6±3.3
Rounds carried out by LDV, number (m±SD)	
Urban rounds	14.6±30.9
Rural rounds	23.6±31
Mixed rounds	22.8±31.7
Round holder	42.3±42.1
The longest distance of round, Km (m±SD)	79.0±39.8
The shortest distance of round, Km (m±SD)	20.0±16.5
Rounds carried out on foot	
Round holder, number (m±SD)	16.1±4.4
The longest distance of round, Km (m±SD)	8.9±8.1
The shortest distance of round, Km (m±SD)	6.3±6.1
Working schedules, number of centers (%)	
regular schedule	84 (95.5)

irregular/alternate schedule	9 (10.2)
night shift	10 (11.4)
staggered hours	37 (42)
Staff management during peak period, number of centers (%)	
flexible worktime	38 (43.2)
use of additional staff<br>among which:	65 (73.9)
temporary workers	38 (43.2)
contract of determined length	59 (67)
subcontractors	16 (18.2)
Objectives of center, number of centers (%)	
existence of individual objectives	79 (89.8)
existence of collective objectives	85 (96.6)
evolution of objectives during the last 2 years	58 (40.6)
among which: evolution towards more demanding objectives	53 (91.4)
Control of workers, number of centers (%)	
existence of operator control at the workstation	69 (48.3)
among which, type of control:	
control exercised by management	56 (81.2)
computer-based monitoring	55 (79.7)
New work organizations, number of centers (%)	
centers concerned by the new work organization	45 (51.18)
among which work organization including a lunchbreak	29 (32.95)
Training courses	
Existence of training adapted to the activities of operators within the center, number of centers (%)	84 (95.5)
Number of training courses completed by employees in the past 5 years, mean (min-max)	
driving training	1.78 (0-5.5)
handling loads training	0.92 (0-3.3)
