# Supplementary material for: Low Back Pain and Upper-Extremity Musculoskeletal Disorders in French Postal Workers Driving Light-Duty Vehicles for Mail and Parcel Delivery
Source: Int J Environ Res Public Health. 2023 Jan 31;20(3):2509. doi: 10.3390/ijerph20032509 (PMC9916289; doi:10.3390/ijerph20032509)
Supplement: Supplementary file 1 [file ijerph-20-02509-s001.zip › Supplementary File S2.pdf]

Supplementary File S2. Organizational factors selected in the backward-stepwise selection (p <0.20) adjusted on personal factors. Results are expressed by a regression coefficient β (p-value) [95% confidence interval]. Abbreviations: LBP: low back pain; UEMSDs: upper-extremity musculoskeletal disorders. (d) organizational factors not selected in the stepwise procedure; (e) model unavailable.

	LBP		UEMSDs	
	men<br>β (p) [95% CI]	women<br>β (p) [95% CI]	men<br>β (p) [95% CI]	women<br>β (p) [95% CI]
**At worker level**				
Observations	214	128	216	127
Concerned by new work organizations (yes vs no)	0.24 (0.050) [-0.00;0.48]	0.36 (0.065) [-0.02;0.74]	0.50 (0.135) [-0.16;1.16]	(d)
Work schedule (vs regular hours)				
Irregular hours	-0.11 (0.690) [-0.66;0.44]	(d)	-1.57 (0.153) [-3.73;0.59]	(d)
Staggered hours	-0.40 (0.060) [-0.82;0.02]	(d)	1.04 (0.028) [0.11;1.96]	(d)
Can take a break (yes vs no)	(d)	(d)	-0.65 (0.128) [-1.49;0.19]	(d)
Round holder (yes vs no)	(d)	(d)	1.46 (0.001) [0.58;2.34]	(d)
Driver training in the past 5 years (yes vs no)	(d)	-0.50 (0.044) [-0.99;-0.01]	(d)	-1.40 (0.016) [-2.54;-0.26]
Automatic gearbox vs. non automatic gearbox	-0.38 (0.199) [-0.96;0.20]	(d)	-0.98 (0.095) [-2.13;0.17]	(d)
Electric/hybrid vehicle vs combustion-powered vehicle	0.40 (0.108) [-0.09;0.88]	(d)	(d)	0.77 (0.199) [-0.40;1.93]
Perceived difficult-to-achieve assigned objectives (yes vs no)	(d)	(d)	(d)	0.73 (0.095) [-0.13;1.58]
Premium in the last 2 years (yes vs no)	(d)	-0.65 (0.072) [-1.35;0.06]	(d)	(d)
Handling loads training in the past 5 years (yes vs no)	(d)	(d)	-0.79 (0.051) [-1.58;0.00]	0.61 (0.184) [-0.29;1.50]
**At center level**				
Observations	111	88	112	88
New work organization (vs no new work organization)				
with lunchbreak	(d)	0.50 (0.067) [-0.04;1.04]	(d)	(e)
without lunchbreak	(d)	0.01 (0.982) [-0.60;0.61]	(d)	(e)
Flexible working time during peak periods	(d)	0.45 (0.069) [-0.03;0.93]	0.72 (0.146) [-0.25;1.69]	(e)
Use of additional staff during peak periods	-0.54 (0.078) [-1.15;0.06]	(d)	-1.16 (0.048) [-2.31;-0.01]	(e)
Evolution towards more demanding objectives during the last 2 years	0.40 (0.069) [-0.03;0.83]	(d)	(d)	(e)
Operators control (computer-based and management-based)	-0.80 (0.002) [-1.31;-0.30]	(d)	(d)	(e)
